# Influence of Phosphomolybdates on Flame Retardancy and Smoke Suppression of PVC Matrix Flame Retardant Composites

**DOI:** 10.3390/molecules30214269

**Published:** 2025-11-03

**Authors:** Xuan Zhou, Zhiyu Hu, Feng Jiang, Quancheng Yang, Ming Gao

**Affiliations:** 1 Hebei Key Laboratory of Hazardous Chemicals Safety and Control Technology, School of Chemical Safety, North China Institute of Science and Technology, Sanhe 065201, China; 2 State Key Laboratory of Biobased Fiber Manufacturing Technology, China Textile Academy, Beijing 100025, China

**Keywords:** phosphomolyptate, Sb_2_O_3_, PVC, flame retardancy, smoke suppression

## Abstract

Four types of phosphomolybdates (iron, cobalt, nickel, and zinc) were each combined with antimony trioxide (Sb_2_O_3_) to prepare polyvinyl chloride (PVC) composites. The effects of the phosphomolybdates on the combustion behavior, smoke release, and mechanical properties of the PVC composites were studied by thermogravimetric analyzer, limiting oxygen index (LOI) tester, smoke density tester, cone calorimeter, scanning electron microscopy (SEM), and universal tensile tester. The results indicate that phosphomolybdates exhibit a significant synergistic flame-retardant effect with Sb_2_O_3_ in the PVC matrix. Compared with Sb_2_O_3_ alone, the addition of phosphomolybdates can significantly improve the LOI of PVC composites. It can also reduce smoke release, lower the heat release rate (HRR) of PVC composites, and produce more char residual, which is more continuous and denser. Meanwhile, there is no significant loss of mechanical properties. Overall, nickel phosphomolybdate has been determined to be the most effective of these four phosphomolybdates. Compared with PVC-1, the peak heat release rate (PHRR) of PVC-4 with nickel phosphomolybdate decreased by 13.3%, the total smoke production (TSP) decreased by 37.3%, and the peak smoke production rate (PSPR) decreased by 28.9%. This study demonstrates that replacing some of the Sb_2_O_3_ with phosphomolybdate can achieve efficient flame retardancy and smoke suppression in PVC. And the results of this study can also provide a reference for future research on the application and promotion of flame-retardant PVC.

## 1. Introduction

Polyvinyl chloride (PVC) is an economical and versatile thermoplastic that is widely used in construction, framing, and decoration due to its excellent mechanical properties, corrosion resistance, plasticity, and insulation capabilities [1,2]. Moreover, pure PVC has a good self-flame-retardant property due to its high chlorine content. However, in order to improve the processing performance of PVC materials, it is often necessary to add a large amount of plasticizers to PVC, such as dioctyl phthalate (DOP) or trioctyl metaphthalate, etc. And it will significantly reduce the limiting oxygen index (LOI) value of PVC and increase the smoke released during combustion [3,4]. Smoke and toxic fumes are the main cause of human deaths in fires, so it is crucial to add flame retardants to PVC composites to improve the flame retardancy while inhibiting smoke release [5,6].

According to a large number of studies, the effective flame retardants and smoke suppressants of PVC mainly involve hydroxides, nanoparticles, antimony trioxide (Sb_2_O_3_), and transition metal compounds, etc. [7,8,9,10,11,12,13,14]. Hydroxides have exhibited significant smoke suppression effects and pollution-free properties. However, to achieve a comparable flame-retardant effect, the required addition level of hydroxides is exceptionally high. This makes it difficult to disperse the hydroxides within the polymer matrix and severely compromises the mechanical properties of the composites [8,9,10]. In recent years, nanoparticles have been extensively studied as flame-retardant additives in PVC. Although nanoparticles can improve the thermal properties and combustion behavior of PVC, they tend to agglomerate readily within the polymer matrix owing to their high surface energy [13,14]. Consequently, surface chemical modification is often required for nanoparticles, making their large-scale application restrictive.

Among these flame retardants, Sb_2_O_3_ has a significant flame-retardant effect due to its excellent synergistic flame-retardant effect with halogens [15]. And this system acts mainly in the gas phase during combustion [16]. However, antimony is a toxic element. When burned, antimony compounds can produce toxic or irritating vapors that are very harmful to human health. Even so, Sb_2_O_3_ has consistently attracted attention due to its outstanding synergistic flame-retardant activity with halogenated flame retardants. So, it is important to develop eco-friendly and efficient flame retardants to replace some or all of the Sb_2_O_3_.

Many studies have shown that most transition metal compounds, such as molybdenum, iron, zinc, cobalt, etc., are effective smoke suppressants [17,18]. In addition, sufficient research has shown that molybdenum can effectively improve the strength and yield of the composite char layer during the combustion. This, in turn, can improve the ability to isolate flames and high temperatures, reduce smoke emissions, and achieve a significant smoke suppression effect [19,20].

In addition, related research has utilized molybdenum trioxide and ammonium octamolybdate, which are commonly used in molybdenum compounds, and ammonium phosphomolybdate to conduct flame-retardant studies on semi-rigid PVC. It was found that all the three compounds could reduce the smoke emission of PVC composites and improve flame-retardant performance, for which ammonium phosphomolybdate was the most prominent [21]. Ammonium phosphomolybdate is a type of phosphomolybdate.

Phosphomolybdate (A[PMo_12_O_40_], A = Fe, Co, Ni, Zn, etc.), where A is usually a transition metal, is obtained by reacting phosphomolybdenum acid (H_3_PMo_12_O_40_) with the relevant transition metal compounds. The molecular formula of phosphomolybdate contains both a flame-retardant element—phosphorus element—and two smoke-suppression elements—molybdenum element and one of the above transition metal elements. Therefore, using phosphomolybdates to improve the flame retardancy and smoke suppression of PVC would be a good choice. Nevertheless, there has been very little research on the use of transition metal phosphomolybdate for flame retardancy in polymers.

In this study, four phosphomolybdates (iron, cobalt, nickel, and zinc) were used separately as flame retardants and smoke suppressants to replace part of Sb_2_O_3_, and PVC composites with different phosphomolybdates were then prepared by melt blending. The combustion behavior of the PVC composites was studied via a LOI tester, a smoke density meter, a thermogravimetric analyzer, cone calorimetry, and SEM. And the effect of phosphomolybdates on the combustion behavior of the PVC composites when combined with Sb_2_O_3_ was investigated, and the underlying flame-retardant and smoke-suppressing mechanisms were also explored.

## 2. Results and Discussion

### 2.1. LOI

The LOI is a direct indicator of the flammability of PVC materials and Table 1 shows the LOI of PVC and its flame-retardant composites. The results show that the LOI of PVC-0, which has not undergone flame-retardant treatment, is only 28.8%. The PVC-1 composite is obtained by adding 6.0 g of Sb_2_O_3_ to PVC-0. The LOI of PVC-1 increases to 34.1%, showing that Sb_2_O_3_ has an obvious flame-retardant effect on PVC. With the same amount of individual phosphomolybdate added, the LOIs of PVC-6, PVC-7, PVC-8, and PVC-9 increased to 30.3%, 29.8%, 29.6%, and 30.0%, respectively. Therefore, phosphomolybdates can also improve the flame retardancy of PVC; however, their contribution to the LOI is significantly less than that of Sb_2_O_3_. Additionally, when the total amount of flame retardant is kept constant and one phosphomolybdate replaces some of the Sb_2_O_3_, the LOI of PVC-2, which contains 4.5 g of Sb_2_O_3_ and 1.5 g of iron phosphomolybdate, increases to 35.2%. This is a 3.23% increase relative to PVC-1. The LOI of PVC-3 with 4.5 g of Sb_2_O_3_ and 1.5 g of cobalt phosphomolybdate increase to 35.1%, which is 2.93% higher than that of PVC-1. The LOI of PVC-4 with 4.5 g of Sb_2_O_3_ and 1.5 g of nickel phosphomolybdate increases by 2.64%, reaching 35.0%. The LOI of PVC-5, which contains 4.5 g of Sb_2_O_3_ and 1.5 g of zinc phosphomolybdate, is the same as that of PVC-3. It also shows a 2.93% improvement over PVC-1. It can be seen that there is an obvious synergistic effect between phosphomolybdates and Sb_2_O_3_, which can effectively improve the LOI of PVC composites.

### 2.2. Smoke Density Tests

The maximum smoke density (MSD) and smoke density rating (SDR) of PVC and its composites are presented in Table 2. The MSD of PVC-0 is 98.5, and the SDR is 88.1. After the addition of Sb_2_O_3_, the MSD of PVC-1 decreases to 95.8, and the SDR decreases to 86.9. This indicates that the addition of Sb_2_O_3_ can reduce both the MSD and SDR of PVC. After the addition of phosphomolybdates, the MSDs of PVC-2, PVC-3, PVC-4, and PVC-5 are further reduced to 94.0, 93.2, 92.6, and 94.5, and the SDRs are also reduced to 83.7, 81.8, 77.9, and 83.9, respectively, showing that the addition of all the phosphomolybdates above can reduce the MSD and SDR of PVC. Compared with PVC-1, the MSD of cobalt phosphomolybdate is reduced by 2.7% and its SDR by 5.9%. The MSD of nickel phosphomolybdate is reduced by 3.4% and its SDR by 10.3%. It suggests that the two phosphomolybdates, cobalt phosphomolybdate and nickel phosphomolybdate, exhibit excellent smoke suppression performance when combined with Sb_2_O_3_.

### 2.3. Cone Calorimeter Test

**Figure 1:**
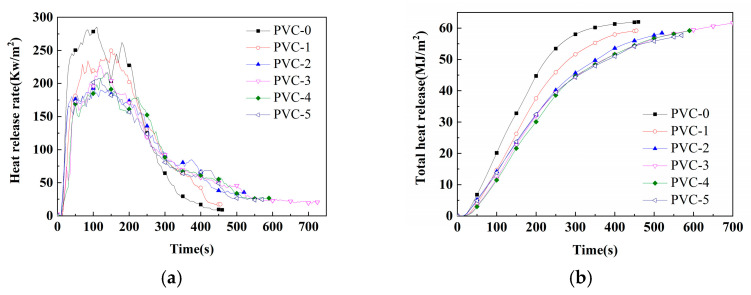
(**a**) HRRs of PVC and its composites; (**b**) THRs of PVC and its composites.

**Figure 2:**
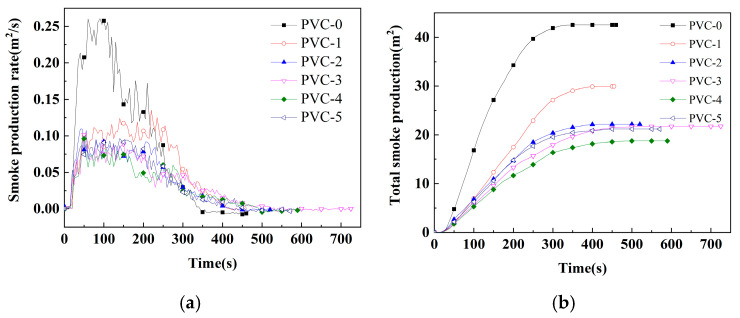
(**a**) SPRs of PVC and its composites; (**b**) TSP of PVC and its composites.

**Figure 3:**
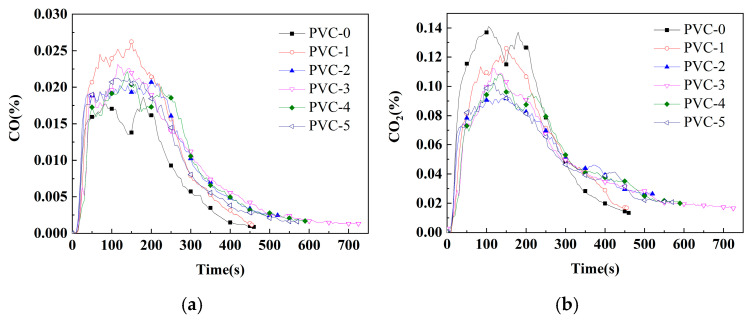
(**a**) CO-yield profile of PVC and its composites; (**b**) CO_2_-yield profile of PVC and its composites.

**Figure 4:**
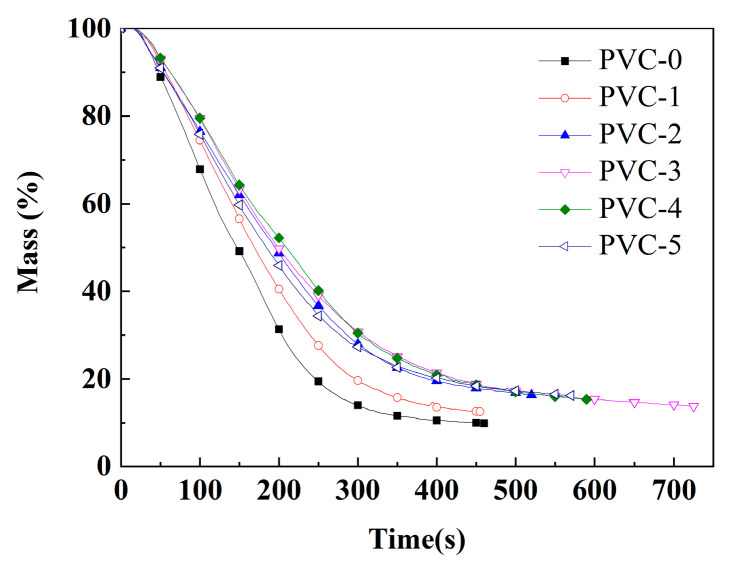
Mass loss of PVC and its composites.

The cone is the ideal instrument for testing the combustion properties of materials by simulating real combustion environments, and its data can provide a more comprehensive evaluation of the real combustion behavior of materials in a fire. Table 3 lists important parameters related to PVC and its composites, including effective heat of combustion (EHC), peak heat release rate (PHRR), time to peak heat release rate (T_PHRR_), total heat release (THR), fire growth index (FGI), fire performance index (FPI), total smoke production (TSP), peak smoke production rate (PSPR), peak carbon monoxide yield (Y_CO_), peak carbon dioxide yield (Y_CO_2__), mean mass loss rate (mean MLR), and char residue rate. Additionally, Figure 1, Figure 2, Figure 3 and Figure 4 show the curves of heat release rate (HRR), THR, TSP, smoke production rate (SPR), carbon monoxide (CO) yield, carbon dioxide (CO_2_) yield, and sample mass over time.

As demonstrated in Figure 1, the PHRR of PVC-0 is 285 kW/m^2^ at 110 s, indicating a substantial thermal and fire risk. Following the incorporation of Sb_2_O_3_, the T_PHRR_ of PVC-1 is postponed to 150 s, and the PHRR exhibits a decline to 250 kW/m^2^. Following the incorporation of phosphomolybdates, the PHRRs undergo a reduction, with the PHRRs of PVC-2, PVC-3, PVC-4, and PVC-5 decreasing to 193 kW/m^2^, 227 kW/m^2^, 216 kW/m^2^, and 208 kW/m^2^, respectively, which correspond to a decrease of 22.8%, 9.2%, 13.6%, and 16.8%, respectively, in comparison with PVC-1. The T_PHRRs_ of PVC-2, PVC-3, PVC-4, and PVC-5 are 100 s, 120 s, 140 s, and 115 s, respectively. The findings indicate that the incorporation of phosphomolybdates leads to a substantial decrease in the PHRRs of PVC composites, with the T_PHRRs_ also exhibiting a reduction, albeit to different extents. Among them, the PHRR of PVC-2 with the addition of iron phosphomolybdate decreased the most significantly, while the T_PHRR_ of PVC-2 decreased by 50 s compared with that of PVC-1. This indicates that the PHRR of PVC-2 will occur earlier, but the PHRR is significantly lower. These results indicate that the synergistic effect between iron phosphomolybdate and Sb_2_O_3_ can best promote the reduction of HRR of the PVC matrix. Furthermore, during the combustion process, the spread and development of the four PVC composites incorporating phosphomolybdates are comparatively slower and less hazardous compared to PVC-0 and PVC-1.

FGI is the ratio of PHRR to T_PHRR_, and lower values indicate a reduced fire risk. As demonstrated in Table 3, the FGI of PVC-0 is 2.6 kW/(m^2^·s), and the FGI of PVC-1 decreased to 1.7 kW/(m^2^·s). This finding suggests that Sb_2_O_3_ can mitigate the fire risk to a certain extent. Subsequent to the incorporation of phosphomolybdates, the FGIs of PVC-2, PVC-3, PVC-4, and PVC-5 are determined to be 1.9 kW/(m^2^·s), 1.9 kW/(m^2^·s), 1.5 kW/(m^2^·s), and 1.8 kW/(m^2^·s), respectively. From the results, only PVC-4 with the addition of nickel phosphomolybdate has shown a reduced fire risk compared with PVC-1. Therefore, PVC-4 is the safest composite and has the lowest fire risk. The addition of nickel phosphomolybdate greatly improves the fire safety of PVC composites. However, PVC composites with iron, zinc, or cobalt phosphomolybdate have an increased fire risk compared with PVC-1.

The THRs of PVC and its composites are shown in Figure 2. Initially, the THR vs. time curve of PVC-0 rises significantly and the THR increases rapidly, indicating that PVC-0 burns quickly. In contrast, the THR of PVC-1 is lower than that of PVC-0. The lower the THR, the less heat is released during combustion and the better the flame retardance of the material. And the THRs of PVC composites containing phosphomolybdates decrease significantly under the same time condition. At the end of combustion, the THR of PVC-0 is as high as 62.0 MJ/m^2^, while the THR of PVC-1 decreases to 59.2 MJ/m^2^. This is mainly due to the flame-retardant effect of Sb_2_O_3_ in PVC. And the THRs of PVC-2, PVC-3, PVC-4, and PVC-5 with the addition of phosphomolybdates are 58.3 MJ/m^2^, 62.1 MJ/m^2^, 59.1 MJ/m^2^, and 57.6 MJ/m^2^, respectively, suggesting that the addition of phosphomolybdates can reduce the HRRs of PVC composites compared with PVC-1. Notably, the THR of PVC-4 with nickel phosphomolybdate develops most slowly over time, indicating a possible significant synergistic effect between nickel phosphomolybdate and Sb_2_O_3_ in the PVC matrix. Additionally, although PVC-3 has a higher THR than PVC-1, its HRR is much slower over time, making the composite relatively safer. Furthermore, the HRR and THR results indicate that adding phosphomolybdates can significantly reduce the THR and HRR, which helps to improve the safety of PVC composites, even though the reduction in THR after the fire is not very obvious.

Smoke has been identified as a primary factor contributing to the high mortality rate associated with conflagrations [22]. Consequently, the reduction in smoke production from materials involved in such conflagrations has emerged as a pivotal research priority, particularly in the context of the development of flame-retardant materials. Among the parameters that must be assessed in order to evaluate the fire hazard are TSP, SPR, Y_CO_, and Y_CO_2__. As demonstrated in Table 3 and illustrated in Figure 3 and Figure 4, the TSP value of PVC-0 is determined to be 42.5 m^2^/m^2^, and the PSPR value of smoke release is found to be as high as 0.26 m^2^/s. Following the incorporation of Sb_2_O_3_, the TSP value of PVC-1 is diminished to 29.9 m^2^/m^2^, signifying a 29.7% reduction compared with PVC-0. And the PSPR of PVC-1 is reduced to 0.14 m^2^/s, which is 48.3% lower compared with PVC-0. This result indicates that Sb_2_O_3_ has a significant inhibitory effect on PVC smoke release. Following the incorporation of phosphomolybdates, the TSP values of PVC-2, PVC-3, PVC-4, and PVC-5 diminish to 22.1 m^2^/m^2^, 21.7 m^2^/m^2^, 18.7 m^2^/m^2^, and 21.2 m^2^/m^2^, respectively. Notably, the addition of nickel phosphomolybdate exhibits remarkable efficacy, as evidenced by the substantial reduction in the TSP value of PVC-4, which is decreased by 37.3% compared with that of PVC-1. Furthermore, the PSPR values of PVC-2, PVC-3, PVC-4, and PVC-5 are 0.11 m^2^/s, 0.10 m^2^/s, 0.10 m^2^/s, and 0.09 m^2^/s, respectively, which are reduced by 18.5%, 25.2%, 28.9%, and 30.4%, respectively, compared with that of PVC-1. It has been demonstrated that any of iron phosphomolybdate, cobalt phosphomolybdate, nickel phosphomolybdate, and zinc phosphomolybdate can markedly reduce the TSP and PSPR of the PVC composites when compounded with Sb_2_O_3_. One possible reason is that the combination of phosphomolybdates and Sb_2_O_3_ can induce the PVC matrix to form a better char layer during combustion. This inhibits the escape of smoke particles generated by the decomposition of PVC composites.

The Y_CO_ of PVC-0 is 0.02%, and the Y_COs_ of PVC-1, PVC-2, PVC-3, PVC-4, and PVC-5 increase to 0.03%, 0.02%, 0.02%, 0.02, and 0.02%, respectively. This indicates that the PVC composites produce more CO following the flame-retardant treatment. Herein, the Y_CO_ of the PVC composites that have been treated with phosphomolybdates lies between the Y_COs_ of PVC-0 and PVC-1. Furthermore, the Y_CO_2__ of PVC-0 is found to be 0.14%, while the Y_CO_2__ of PVC-1 decreases to 0.13%. This observation suggests that the incorporation of Sb_2_O_3_ leads to incomplete combustion of the material, resulting in an increase in Y_CO_ and a decrease in Y_CO_2__ compared with PVC-0. Moreover, the Y_CO_2_s_ of PVC-2, PVC-3, PVC-4, and PVC-5 decrease to 0.09%, 0.11%, 0.11%, and 0.10%, respectively, compared with that of PVC-1. The findings suggest that the incorporation of phosphomolybdates lead to the inhibition of both Y_CO_ and Y_CO_2__, a phenomenon that is not observed when Sb_2_O_3_ is added alone, resulting in a decrease in final gas production. Thus, the combined action of phosphomolybdates and Sb_2_O_3_ can reduce the spread of fire, leading to incomplete combustion of PVC along with a reduction in the production of the two toxic gases.

In addition, the mean MLRs of PVC-0 and PVC-1 are 0.10 g/s and 0.10 g/s, respectively. The mean MLRs of PVC composites are reduced to 0.08 g/s, 0.06 g/s, 0.07 g/s, and 0.07 g/s, respectively, after the addition of phosphomolybdates. These results indicate that the incorporation of phosphomolybdates reduces the mass loss of the PVC composites. As illustrated in Figure 4, the mass loss of PVC-4 with the addition of nickel phosphomolybdate prior to 300 s is the least, and the composite exhibits optimal stability. Subsequently, the mass loss of PVC-3 with the addition of cobalt phosphomolybdate becomes the least. Correspondingly, the char residual rate of PVC-0 is only 9.9%, indicating a poor charring ability of pure PVC. The char residual rate of PVC-1 after combustion is 12.5%, which is 21.3% higher than that of PVC-0, indicating that the addition of Sb_2_O_3_ could promote the charring of the PVC matrix. Following the addition of phosphomolybdates, the char residual rates of PVC-2, PVC-3, PVC-4, and PVC-5 are 16.3%, 13.8%, 15.3%, and 16.2%, respectively. In comparison with PVC-1, there is an increase of 30.2%, 9.8%, 22.4%, and 29.6%, respectively. This indicates that replacing Sb_2_O_3_ with an appropriate amount of phosphomolybdates has a significant effect on promoting the charring of PVC substrate, and the resulting char layer can effectively inhibit the release of smoke and toxic gases.

### 2.4. Thermal Degradation

**Figure 5:**
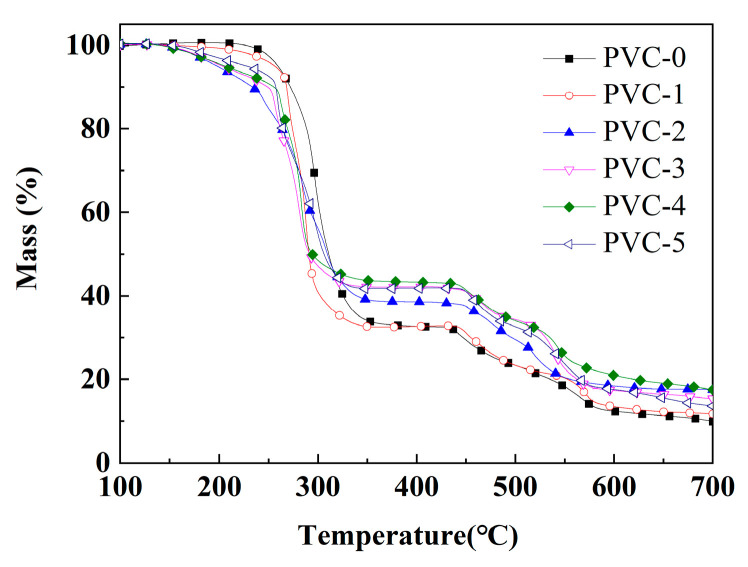
Thermogravimetric curves of PVC and its composites.

The thermogravimetric curves of pure PVC and its composites are shown in Figure 5, and the related parameters are listed in Table 4. T_−20%_ represents the temperature corresponding to 20% mass loss. T_max1_, T_max2,_ and T_max3_ represent the temperatures corresponding to the maximum degradation rates of the first, second, and third degradation stages, respectively. As demonstrated by the curves in Figure 5, the thermal degradation of the composites is divided into three stages as the temperature increases [23]. The mass loss observed in the initial stage (200–350 °C) is predominantly attributed to the degradation of the plasticizer DOP and the release of hydrogen chloride (HCl) from PVC [24]. In the second stage (350–500 °C), the mass loss is primarily due to the PVC molecular chains being further cleaved and degraded after the removal of HCl, leading to cross-linking and carbonization [25]. The mass loss in the third stage (500–700 °C) is mainly due to the volatilization of aromatic compounds formed by cyclization within the conjugated polyene molecules with a further increase in temperature [26].

As shown in Table 4, the T_max1_ of PVC-0 in the initial stage is 297 °C, while the T_max1_ of PVC-1 following the incorporation of Sb_2_O_3_ exhibits a decline to 286 °C. It has been demonstrated that Sb_2_O_3_ facilitates the degradation of the PVC composites, and, as a metal oxide, it promotes the removal of HCl and the degradation of DOP in PVC [27]. With the further addition of phosphomolybdates, it has been demonstrated that the T_max1_ of the PVC composites continued to decrease compared with PVC-1. Also, the T_−20%_ of the PVC-0 composite is 289 °C, and the addition of Sb_2_O_3_ reduces the T_−20%_ of PVC-1 to 274 °C. Similarly, the addition of phosphomolybdates continues to reduce the T_−20%_ of the PVC composites compared with PVC-1. It is hypothesized that phosphomolybdate, as a metal salt, can catalyze the removal of HCl from PVC. However, the T_max2_ of PVC composites at the second degradation stage is significantly higher than that of PVC-0 after the addition of the flame retardant, and the T_max2_ of PVC composites with the addition of phosphomolybdates is even higher than that of PVC-1, which suggests that the incorporation of phosphomolybdates promotes cross-linking and charring in the early stage of PVC. As illustrated in Table 4, the T_max2_ of PVC-2 with iron phosphomolybdate is 473 °C, which is the highest among all the PVC composites, indicating that this promoting effect of iron phosphomolybdate is the most obvious. In addition, the findings in Figure 4 indicate that PVC-4 with nickel phosphomolybdate exhibits optimal thermal stability, while PVC-2 with iron phosphomolybdate demonstrates the weakest thermal stability among the PVC composites with phosphomolybdates in the second stage. In addition, in the third stage, the T_max3_ of PVC-0 is 566 °C and that of PVC-1 is also 566 °C. However, the T_max3_ of the PVC composites decreased after the addition of iron phosphomolybdate, which suggests that iron phosphomolybdate facilitated the early occurrence of the intramolecular cyclization reaction. Among them, PVC-2, which contains iron phosphomolybdate, exhibits the lowest T_max3_ value of 523 °C. This finding suggests that PVC-2 may be the first to undergo the cyclization reaction during the late stage of pyrolysis due to the addition of iron phosphomolybdate, thus obtaining more char layers. Eventually, the char residual of PVC-0 is 9.93%, and the addition of Sb_2_O_3_ increases the char residual of PVC-1 to 11.70%, and after the addition of phosphomolybdates, the char residuals of PVC-2, PVC-3, PVC-4, and PVC-5 is increased to 17.49%, 15.34%, 17.51%, and 13.61%, respectively, which shows that the incorporation of phosphomolybdates can promote the char formation of PVC composites, with nickel phosphomolybdate exhibiting the most significant effect. These results indicate that the use of Sb_2_O_3_ and phosphomolybdates in combination has a significant effect in accelerating the removal of HCl from PVC, promoting the early cross-linking of the PVC matrix into the char residual and improving the stability of the char residual.

### 2.5. Morphology of Char Residual

**Figure 6:**
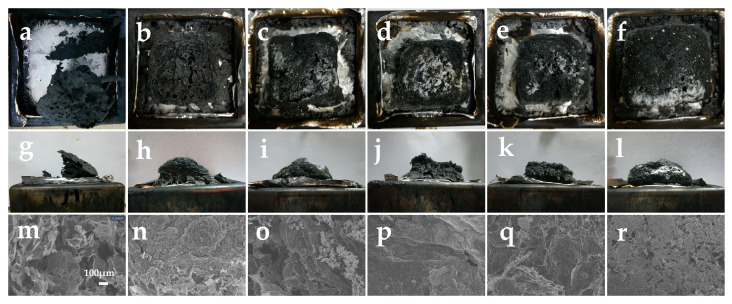
Digital photos and SEM (×100) images of char residual after the cone calorimeter test. Herein, (**a**–**f**) are the top view of digital photos of PVC-0, PVC-1, PVC-2, PVC-3, PVC-4, and PVC-5; (**g**–**l**) are the side view of PVC-0, PVC-1, PVC-2, PVC-3, PVC-4, and PVC-5; and (**m**–**r**) are SEM images of PVC-0, PVC-1, PVC-2, PVC-3, PVC-4, and PVC-5.

Shown in Figure 6a–e,g–k are the frontal and lateral morphologies of the char residual after the cone calorimetry tests, and in m-q are the SEM images (100×) of the char residual, which were used to evaluate the macroscopic and microscopic morphology of the char residual outer surface of PVC and its composites, respectively.

From Figure 6a,g,m, it can be seen that there are still some char residuals remaining in PVC-0 after the cone calorimetric test, but the morphology of this char layer is loose and incomplete, and, likewise, its microstructure is the most irregular and inhomogeneous, with a variety of defects evident on the surface, showing a porous and loose structure. It is difficult for such a char layer to withstand the ingress of external flames and heat. Following the incorporation of Sb_2_O_3_, the char layer of PVC-1 becomes complete, while a large number of holes and cracks can be seen on the surface of the char layer, both on its front and side sides, and the presence of a large number of loose structures can also be seen from the microscopic morphology in Figure 6n. This suggests that the addition of Sb_2_O_3_ has enhanced the quality of the char layer, improving its resistance to flames and heat, albeit only slightly. With the addition of phosphomolybdates, the surfaces of the char layers of PVC composites become glossy and the density of the char layers are large compared with that of PVC-1. The higher the density of the char layer, the better the barrier effect. However, in contrast, the surface of the char layer of PVC-2 with iron phosphomolybdate still has a large number of holes, and these holes are the most numerous in PVC-2 among all the PVC composites with phosphomolybdates, both from the macroscopic and microscopic electron microscope patterns. The char layer is also insufficiently robust to provide protection against external flame erosion. This is why, although PVC-2 forms a relatively large amount of char layer in the later stages of combustion, the quality of the char residual is inadequate, resulting in a relatively large amount of heat as well as flue gases produced by PVC-2 during combustion. In addition, the pores on the char layer surfaces of PVC-3 and PVC-4 with cobalt phosphomolybdate and nickel phosphomolybdate, respectively, are noticeably fewer. From the microscopic morphology of PVC-3, Figure 6p, it can be observed that although there are a few cracks, the surface of the char layer is relatively dense on the whole. Especially in Figure 6q, PVC-4, where it can be clearly observed that the char layer is continuous and tight, and the holes almost disappear. Such a char layer is most effective at inhibiting heat transfer and isolating oxygen from the air; thus, it can protect the PVC matrix and prevent the composite from continuing to burn. As a result, PVC-4 performs well in all PVC composites with phosphomolybdates. Further, the char residual of PVC-5 with zinc phosphomolybdate addition is not dense enough in local areas and some holes existed, which can also be seen in Figure 6r, where some holes are also distributed on the surface of the char layer of PVC-5, but the morphology of the char layer is more solid. The aforementioned results indicate that this synergistic effect between phosphomolybdates and Sb_2_O_3_ can not only promote the increase in the amount of char layer in PVC composites but also promote the density and quality of the char layer. And the performance of nickel phosphomolybdate is the most outstanding, which is consistent with the results of cone calorimetry.

### 2.6. Proposed Flame-Retardant Mechanism

Based on the above experimental results and discussions, a flame-retardant mechanism involving Sb_2_O_3_ and phosphomolybdates is proposed for PVC. During the initial stage of combustion, Sb_2_O_3_ decomposes rapidly and reacts with some of the HCl released from PVC, forming SbCl_3_ [28]. This SbCl_3_ (g) has a high vapor density and can capture free radicals in the gas-phase combustion zone, thereby suppressing the flame and reducing the flame density [29]. Simultaneously, the remaining HCl is released into the gas phase, diluting the oxygen concentration [30]. Sb_2_O_3_ is renowned for its excellent gas-phase flame retardancy, but it produces harmful gases and has a weaker charring ability in the condensed phase. After compounding with phosphomolybdates, the generated HCl can react with the phosphomolybdates to form P-containing oxygen acids, water, molybdates, and transition metal chlorides. Transition metal chlorides of FeCl_3_, C_O_Cl_2_, NiCl_3_, and ZnCl_2_ are all Lewis acids. In the early stages of combustion, the action of Lewis acids promotes the dehydrochlorination of PVC, producing more HCl, which then participates in the reaction again, playing a self-catalytic role [30]. And molybdates also catalyze the dehydrochlorination of PVC through the Lewis acid mechanism [31]. The resulting conjugated olefin chains from the decomposition of PVC can quickly crosslink to form a dense, stable char layer in the presence of Lewis and P-containing oxygen acids at high temperatures, and the layer can serve as an effective barrier to isolate heat and oxygen [32,33]. Therefore, combining phosphomolybdates and Sb_2_O_3_ enable the PVC composites to exhibit dual flame-retardant effects from the gas and condensed phases during combustion. This reduces the release of heat and smoke from the composite, ultimately achieving excellent flame-retardant and smoke-suppression effects. Additionally, it is worth noting that nickel phosphomolybdate has the most prominent promotional effect of the four phosphomolybdates.

### 2.7. Mechanical Properties

Tensile strength and elongation at break are important mechanical property parameters for composites. These two parameters of PVC and its composites are shown in Table 5. With the exception of PVC-0, the total amount of flame-retardant additives in each PVC composite is the same, accounting for about 3.99% of the total mass. The results show that the tensile strength of PVC-0 is 20.54 MPa and that the elongation at break can reach 288%. When Sb_2_O_3_ is added, the tensile strength and elongation at break of PVC-1 increase to 21.32 MPa and 324%, respectively. Clearly, a small amount of a single inorganic flame retardant can improve the mechanical properties of the blends due to the excellent mechanical properties of the retardant itself [15]. After the addition of phosphomolybdates, the tensile strength of PVC-2, PVC-3, PVC-4, and PVC-5 are 19.89 MPa, 20.17 MPa, 19.64 MPa, and 19.68 MPa, respectively, and the elongation at break are 278%, 282%, 269%, and 272%, respectively. It can be seen that after partial substitution of Sb_2_O_3_ by phosphomolybdates, the tensile strength and elongation at break of PVC composites will decrease, but the decrease is minimal. In summary, introducing a small amount of phosphomolybdates did not significantly affect the mechanical properties of the PVC composites, but improved their flame-retardant properties.

## 3. Materials and Methods

### 3.1. Materials

PVC resin was purchased from Beijing second chemical factory (Beijing, China). DOP was industrial grade and supplied by Shanghai East chemical plant (Shanghai, China). Sb_2_O_3_, Zinc stearate, lead sulfate tribasic (LST), and dibasic lead phosphate (DLP) were industrial grade and supplied by Beijing Yili Fine Chemicals Co., Ltd. (Beijing, China). Iron phosphomolybdate, zinc phosphomolybdate, nickel phosphomolybdate, and cobalt phosphomolybdate were all industrial grade and provided by Hangzhou Yuhao Chemical Technology Co., Ltd. (Hangzhou, China). All additives were used as supplied.

### 3.2. Preparation of PVC Composites

All samples were obtained by physical blending as follows: 100 phr PVC resin was mixed with 40 phr plasticizer (DOP), 2 phr stabilizer (LST), 2 phr stabilizer (DLP), 0.5 phr lubricant (zinc stearate), and a certain amount of flame retardant, of which its specific formula is shown in Table 6. The raw materials were mixed at 150 °C for 10 min and compressed at 160 °C under 10 MPa for 8 min. Then the sample was taken out for natural cooling and cut into the test specimens.

### 3.3. Characterization

The limiting oxygen index (LOI) was measured using a JF-3 oxygen index tester (NanjingJiangning Analytical Instrument Factory, Nanjing, China) according to the ASTM D2863-12 standard [34]. The specimen size for the LOI test was 100.0 × 6.5 × 3.0 mm^3^, and the LOI of each sample was the average value obtained from three repeated measurements.

The heat release and smoke emission of PVC samples were tested using a cone calorimeter of FTT 0007 (Fire Testing Technology, West Sussex, UK) according to the ISO 5660-1 standard [35], and each specimen with the dimension of 100 × 100 × 3 mm^3^ was exposed to a heat flux of 50 kW/m^2^.

A General Model JCY-2 instrument (Nanjing Jiangning Analysis Instrument Factory, Nanjing, China) was used to measure the maximum smoke density (MSD) and the smoke density rating (SDR) values according to ASTM D2843 [36]. The specimen size for the smoke density tests was 25.4 × 25.4 × 4 mm^3^, and each specimen was repeated for three times.

Thermogravimetric analysis was recorded by a HCT-2 thermal analyzer (Beijing Hengjiu Scientific Instrument Factory, Beijing, China) under air atmosphere with a heating rate of 10 °C/min from 100 °C to 700 °C.

The char layer formed after the cone calorimeter test was first sputter-coated with a layer of gold and then observed using a SEM of KYKY-EM3200 (Beijing Zhongke KeYi Co. Ltd., Beijing, China) with an accelerating voltage of 20 kV.

The mechanical property testing of PVC composite was conducted using a CMT4204 electronic universal material testing machine (Meters Industrial Systems Co., Ltd., Shanghai, China) according to ISO 527-2 [37]. The tensile rate was set at 20 mm/min, with each specimen measuring 100 × 70 × 1 mm^3^. Each specimen underwent five repeated measurements to obtain an average value.

## 4. Conclusions

In this study, four phosphomolybdates—iron phosphomolybdate, cobalt phosphomolybdate, nickel phosphomolybdate, and zinc phosphomolybdate—were used as partial substitutes for Sb_2_O_3_, respectively, to obtain four PVC composites and PVC-1 with Sb_2_O_3_ alone. It was found that the introduction of phosphomolybdates could increase the LOI of PVC composites significantly under the same amount of flame retardant. The findings of the cone calorimeter test indicated that all the four phosphomolybdates have the capacity to markedly enhance the flame retardancy and smoke suppression properties of PVC composites. In comparison with PVC-1, the PHRRs of PVC-2, PVC-3, PVC-4, and PVC-5 decreased by 22.8%, 9.1%, 13.3%, and 16.7%, respectively. And PVC-4 had a 37.3% reduction in TSP and a 28.9% reduction in PSPR, which were consistent with the smoke density test results. Thermogravimetric analysis and SEM results demonstrated that the addition of phosphomolybdates could enhance the production of char residue, resulting in a char layer that was more continuous and denser. This was particularly evident in the char layers of PVC-3 and PVC-4, which contained nickel phosphomolybdate and cobalt phosphomolybdate, respectively. It was only such a char layer that could effectively isolate oxygen and heat, thereby providing robust protection for the PVC matrix. Furthermore, the significant improvement in the flame-retardant and smoke-suppressing properties of the PVC composites was primarily attributed to the incorporation of phosphomolybdates, which compensated for the inadequate flame-retardant performance of Sb_2_O_3_ in the condensed phase. The presence of phosphomolybdates and Sb_2_O_3_ resulted in a discernible synergistic flame-retardant effect within the PVC matrix. Additionally, the addition of phosphomolybdates had a minimal impact on the mechanical properties of the PVC composites.

## Figures and Tables

**Table 1:** LOIs of PVC and its composites.

Sample	LOI/%
PVC-0	28.8 ± 0.2
PVC-1	34.1 ± 0.1
PVC-2	35.2 ± 0.1
PVC-3	35.1 ± 0.2
PVC-4	35.0 ± 0.1
PVC-5	35.1 ± 0.2
PVC-6	30.3 ± 0.1
PVC-7	29.8 ± 0.1
PVC-8	29.6 ± 0.1
PVC-9	30.0 ± 0.2

**Table 2:** MSDs and SDRs of PVC and its composites.

Sample	MSD	SDR
PVC-0	98.5	88.1
PVC-1	95.8	86.9
PVC-2	94.0	83.7
PVC-3	93.2	81.8
PVC-4	92.6	77.9
PVC-5	94.5	83.9

**Table 3:** The characteristic data tested by cone calorimeter *.

Samples	PVC-0	PVC-1	PVC-2	PVC-3	PVC-4	PVC-5
EHC/MJ·kg^−1^	49.4	57.4	77.6	78.0	66.4	69.6
TTI/s	18	17	16	16	16	16
PHRR/kW·m^−2^	285	250	193	227	216	208
T_PHRR_/s	110	150	100	120	140	115
FGI/(kW/(m^2^·s))	2.6	1.7	1.9	1.9	1.5	1.8
FPI/(s/(kW·m^−2^))	0.06	0.07	0.08	0.07	0.07	0.08
THR/MJ·m^−2^	62.0	59.2	58.3	62.1	59.1	57.6
Y_CO_2__/%	0.14	0.13	0.09	0.11	0.11	0.10
Y_CO_/%	0.02	0.03	0.02	0.02	0.02	0.02
TSP (m^2^/m^2^)	42.5	29.9	22.1	21.7	18.7	21.2
PSPR (m^2^/s)	0.26	0.14	0.11	0.10	0.10	0.09
mean MLR (g/s)	0.10	0.10	0.08	0.06	0.07	0.07
Residues/%	9.9	12.5	16.3	13.8	15.3	16.2

* The variations of FPI, Y_CO_2__, PSPR, mean MLR in Table 3 are all less than 10% and the variations of the others are all less than 5%.

**Table 4:** Thermogravimetric analysis data of PVC and its composites.

Samples	T_−20wt%_ (°C)	T_max1_ (°C)	T_max2_ (°C)	T_max3_ (°C)	700 °C (wt%)
PVC-0	289	297	445	566	9.93
PVC-1	274	286	452	566	11.70
PVC-2	263	281	473	523	17.49
PVC-3	262	277	461	537	15.34
PVC-4	269	278	456	542	17.51
PVC-5	264	275	458	547	13.61

**Table 5:** Tensile strength and elongation at break of PVC and its composites.

Sample	Tensile Strength (MPa)	Elongation at Break (%)
PVC-0	20.54 ± 0.37	288 ± 9
PVC-1	21.32 ± 0.52	324 ± 10
PVC-2	19.89 ± 0.28	278 ± 8
PVC-3	20.17 ± 0.16	282 ± 8
PVC-4	19.64 ± 0.33	269 ± 9
PVC-5	19.68 ± 0.42	272 ± 14

**Table 6:** Composition of PVC and its composites.

Samples	PVC (g)	Sb_2_O_3_ (g)	Iron Phosphomolybdate (g)	Cobalt Phosphomolybdate (g)	Nickel Phosphomolybdate (g)	Zinc Phosphomolybdate (g)
PVC-0	100					
PVC-1	100	6.0				
PVC-2	100	4.5	1.5			
PVC-3	100	4.5		1.5		
PVC-4	100	4.5			1.5	-
PVC-5	100	4.5				1.5
PVC-6	100		6.0			
PVC-7	100			6.0		
PVC-8	100				6.0	
PVC-9	100					6.0

## Data Availability

No new data were created or analyzed in this study. Data sharing is not applicable to this article.
